# Changes in peripheral blood immune cell composition in osteoarthritis

**DOI:** 10.1016/j.joca.2015.06.018

**Published:** 2015-11

**Authors:** F. Ponchel, A.N. Burska, E.M.A. Hensor, R. Raja, M. Campbell, P. Emery, P.G. Conaghan

**Affiliations:** Leeds Institute of Rheumatic and Musculoskeletal Medicine & NIHR-Leeds Musculoskeletal Biomedical Research Unit, Leeds University of Leeds, Leeds, UK

**Keywords:** OA, Blood cell composition, Cell subsets/phenotype, Ageing

## Abstract

**Objectives:**

Immune age-related abnormalities may synergise with osteoarthritis (OA) pathology. We explored whether abnormalities in the blood immune cell composition are present in OA, beyond defects typically associated with ageing.

**Design:**

Blood was collected from 121 healthy controls (HC) and 114 OA patients. Synovial biopsies were obtained from another 52 OA patients. Flow cytometry was used to establish the frequencies of lineage subsets, naïve, memory and regulatory T and B-cells, cells with an abnormal phenotype related to inflammation (IRC) and memory-like CD8^+^ T-cells. Multivariate analysis of covariance (MANCOVA) was used to determine whether the relative subset frequencies differed between HC and OA, controlling for age.

**Results:**

Expected histology and T/B-cell infiltration were observed.

Following age adjusted analysis, we confirmed the lack of age association in HC for CD4^+^, B, NK and NKT cells but a negative trend for CD8^+^ T-cells. In OA, CD4^+^ T-cell and B-cell frequency were lower compared to HC while CD8^+^ T-cell frequencies were higher. CD8^+^ memory-like cells were more likely to be found in OA (odds ratio = 15). Increased CD8^+^ IRC frequencies were also present in OA. The relationship between age and CD4^+^ or CD8^+^ naïve T-cells in HC were changed in OA while the age relationships with memory cells were lost. The increase in CD4^+^ Treg with age was also lost in OA. B-cells showed limited evidence of disturbance.

**Conclusions:**

Immune dysfunction may be present in OA beyond what appears related to ageing; this requires further investigation.

## Introduction

Ageing is a complex phenomenon involving several human systems, simultaneously interacting at different levels^1^. It affects cells, tissues and whole organs; it diminishes homeostasis and increases vulnerability^2^. Many pathways, including the immune system (IS), have been shown to be involved in ageing and age-related diseases^3^, ^4^. In the very elderly, infectious disease is the primary cause of death, highlighting the importance of the adaptive IS. Acquired immune responses decline with age, with poor responses to vaccination, increased susceptibility to infection, higher prevalence of certain cancers associated with viral infection or emerging pathogens^5^, ^6^. In addition, there is increased intrinsic difficulty in dealing with common pathogens, notably herpes zoster or cytomegalovirus (CMV). Paradoxically, this decline is accompanied by an increase in auto-reactivity and the generation of autoantibodies^7^, ^8^. In addition, a wide range of etiological factors contribute to increased low-grade inflammation (known as inflammageing) including chronic antigenic burden, decreased production of sex steroids, subclinical disorders such as atherosclerosis, asymptomatic bacteriuria, as well as cellular senescence of immune cells^3^, ^4^, ^6^. The inflammatory response in ageing therefore represents a major potential contributor to age-related conditions.

Osteoarthritis (OA) is strongly age-related^9^, with evidence of radiographic OA highly prevalent in individuals over the age of 60, though clinically symptomatic OA occurs in a smaller percentage^10^. Many of the age-related and inflammation-prone pathways may be involved in OA. Certain abnormalities of the IS including change in T-cell reactivity^11^, development of autoantibodies^12^, telomere shortening^13^ and activation of inflammatory mechanisms^14^ have been reported in OA^15^. Biomechanical stresses have been proposed as a source of immune stimulation in OA^16^, ^17^ and another hypothesis proposes that collagen breakdown reveals neo-antigens leading to T/B-cell responses^18^. Products of cartilage/bone degradation promote persistent low grade inflammation and synovitis both increasingly recognised as important symptoms of OA^19^. Innate immune responses to calcium crystal deposition have also been reported to activate the inflammasome pathway^20^.

We hypothesized that the complex interplay between the musculoskeletal system and IS may be in part responsible for the development of the clinical syndrome of OA. The aims of this study were therefore to determine if abnormalities in the blood immune cell composition were associated with OA, beyond the defects related to age. We investigated lineage composition for CD4^+^ and CD8^+^ T-cells, B-cells, NK-cells and CD56^+^ T-cells (subsequently referred to as NKT-cells) and phenotyped T-cells and B-cells further for naïve, memory and regulatory subsets as well as for two other cell populations: IRC and memory-like cells.

## Materials and methods

### Patients

Blood samples (EDTA 4 ml) were collected from 121 healthy controls (HC) and 114 patients with OA, and arthroscopically-obtained synovial tissue biopsies from a separate 52 OA patients. The studies were approved by the local Research Ethics Committee and all participants gave written an informed consent.

The HC were recruited from Leeds University staff to specifically include a wide range of age in order to establish reference ranges for healthy ageing. Particular attention was given to excluding HC with any joint symptoms.

The OA blood samples were collected from patients recruited from two main sources. Firstly patients from primary and secondary care clinics were recruited into clinical studies where ACR OA clinical criteria were required for inclusion (*n* = 74); a baseline blood specimen was obtained in these participants. Secondly patients in a secondary care orthopaedic clinic who had been referred for joint replacement and had radiographic OA were sampled (*n* = 40). The included patients had OA at different anatomical sites, with the majority having knee OA but over half having OA in at least two anatomical locations (Table I provides detailed numbers). Demographic and clinical characteristics were recorded in the OA patients: age, sex, height and weight, calculated BMI and anatomical location of joint(s) affected by OA (summarised in Table I).

Synovial tissue samples were obtained from patients with ACR clinical OA and evidence of cartilage loss during knee arthroscopy, using 3.5 mm grasping biopsy forceps under direct vision using a Hopkins 2.7 mm 300 arthroscope. A Visual Analogue Score (VAS) was used as a parameter of macroscopic joint inflammation. Scoring was based on the visual inspection of vessels and hyperemia in the synovial membrane^21^. Synovial biopsies were taken from areas of macroscopic synovitis. Age and sex were recorded for these patients (Table I).

### Tissue sample processing

Tissue for light microscopy was fixed in 10% neutral buffered formalin. Sections were then dewaxed in xylene and ethanol, then washed in water and stained with Mayers Haematoxylin and counterstained in 1% aqueous Eosin Y after which they were dehydrated in ethanol cleared in xylene and mounted in DPX.

The paraffin embedded sections were dewaxed in Access Super solution (Menarini diagnostics) then stained. Staining was performed using standard X-Cell plus staining kit (Menarini diagnostics) with modification according to individual primary antibody host species according to manufacturer's instructions. Incubation were with primary monoclonal antibody anti CD3 (rabbit monoclonal clone SP7 Abcam cat ab16669 (1:200), anti CD68 (Rat monoclonal Abcam ab53444, 1:100) and anti CD20 (M-20 Goat polyclonal Santa Cruz sc-7735, 1:200)). The slides were then counterstained in haematoxylin.

### Flow cytometry

8-colours flow-cytometry was performed using standard protocols for cell surface and intracellular staining to establish the frequencies of: CD4^+^ and CD8^+^ T-cells (CD3^+^CD4^+^ and CD3^+^CD8^+^), B-cells (CD19^+^), NK (CD56^+^CD3^-^) and NKT-cells (CD56^+^CD3^+^). CD4^+^ and CD8^+^ T-cells with a naïve (CD45RB^high^CD45RA^+^CD62L^+^) and memory (CD45RB^lowh^CD45RA^−^CD62L^−^) phenotypes were subsequently analysed as well as cells with an abnormal phenotype (Inflammation Related Cells, IRC: CD45RB^high^ CD45RA^+^CD62L^−^) identified in inflammatory arthritis in relationship with CRP^22^. An additional CD8^+^ T-cell population with a memory-like phenotype (CD45RB^low^CD45RA^−^CD62L^+^) was also present in some but not all samples (see Supplementary Figures). Regulatory T-cells (CD3^+^CD4^+^ CD25^high^ Foxp3^+^CD127^low^) were identified in a separate panel. B-cell analysis included a phenotyping panel for naïve (CD19^+^CD27^−^CD38^high^), memory (CD19^+^CD27^+^CD38^low^), and putative regulatory-B-cells defined as transitional T2 cells (CD19^+^CD38^high^CD24^high^), which are known to include regulatory B-cells in human even if not only those, the intracellular expression of IL-10 remaining the defining feature of Breg. A population of Pre-plasma cells (PPC: CD19^+^CD27^+^CD38^high^) could be identified in some sample but not all. All antibody clones used are described in Supplementary file Table S1 with gating strategies exemplified in Figure S1 (A, B, C, D).

### Statistical analysis

Comparison between tissue architecture groups in synovial biopsies was performed using Mann–Whitney *U*-test and Spearman's correlation coefficient.

Following appropriate transformation of variables where necessary to an approximate Gaussian distribution, we used multivariate analysis of covariance (MANCOVA) to determine whether the relative subset frequencies differed between HC and OA, controlling for age. This approach was chosen to account for the inter-relationships between frequencies therefore we ran separate models for lymphocyte lineages (including NK, NKT, CD8^+^ T-cells, CD4^+^ T-cells, B-cells), CD4^+^ T-cell phenotypes (naïve, memory and IRC), CD8^+^ T-cell phenotypes (as for CD4^+^) and B-cells (naïve, memory and putative T2/Breg). Age-adjusted between-groups differences were first calculated assuming the association with age was the same in both groups. An interaction term between age and group was then added to verify whether this assumption was reasonable. If the interaction was significant at the 10% level, changes with age were presented separately for HC and OA.

In the case of Treg cells, linear regression was used, according to the same analysis strategy. Memory-like CD8^+^ T-cells and PPC were analysed separately as not detected in all samples, first using logistic regression to identify age-adjusted between-group differences in the odds of them being present, followed by linear regression restricted to patients in whom the cells were detected. Analyses were conducted in SPSS 21.0.0.1 and Stata 13.1.

## Results

### Synovial tissue

In 26 of the OA synovial tissue biopsies, a VAS was available and ranged from 3 to 80 with a median at 35, suggesting quite high inflammatory scores. Synovitis was evaluated using a scoring system^21^, ^23^ utilising thickness of the lining layer, infiltration of immune cells in the stromal compartment of the synovial membrane and their higher organisation in aggregates or germinal centre-like structures. The score ranged from 0 to 8 with a median of 2.5. VAS was directly associated with the synovitis score (*rho* = +0.493, *P* = 0.012).

**Fig. 1:**
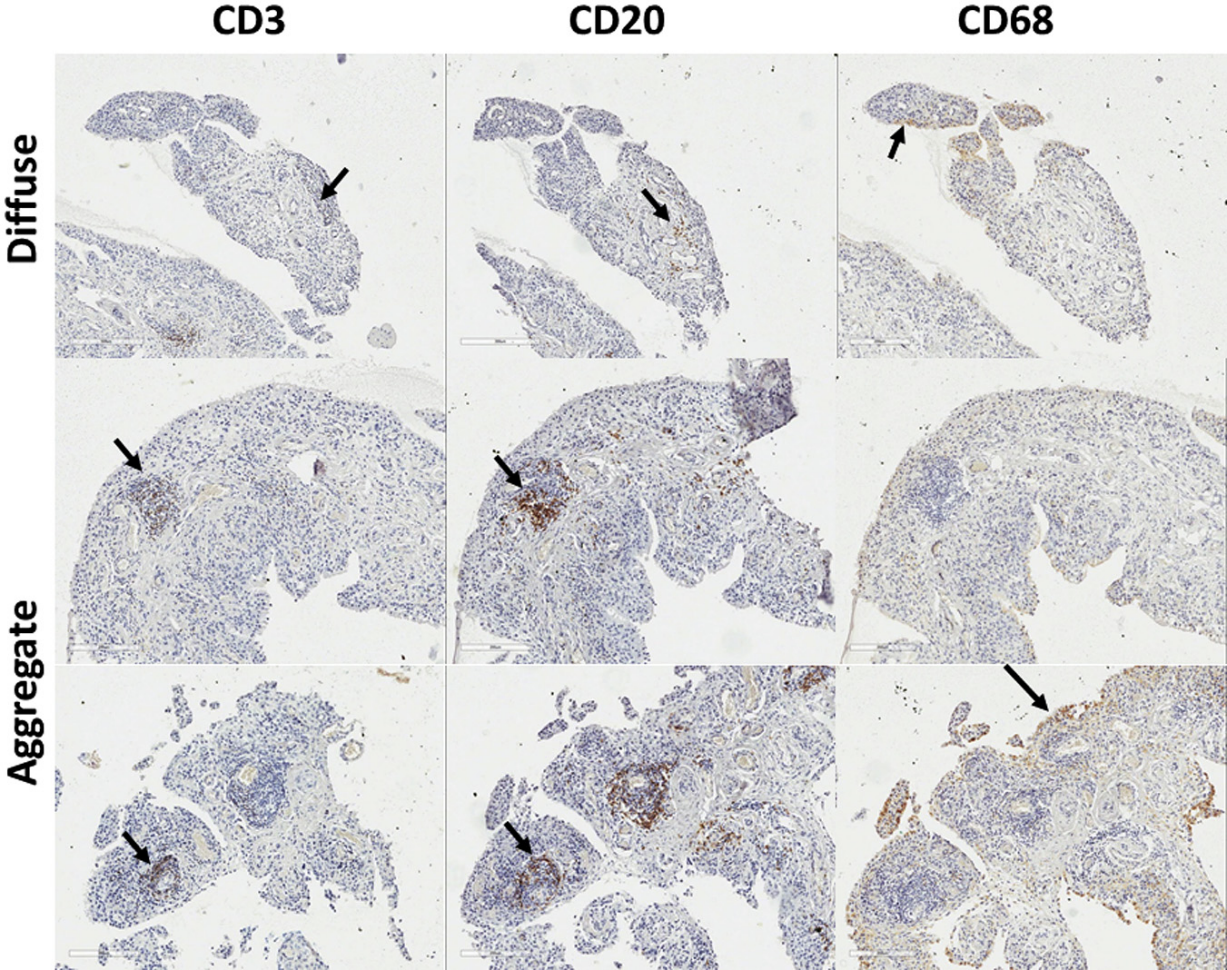
Lineage cell detection in OA synovial tissue. IHC for T-cells (CD3) B-cells (CD20) and macrophage (CD68) in three typical OA biopsies with diffuse, aggregates or germinal centre like structure tissue architecture.

In 13 samples, the histological appearance of the tissue was normal (synovitis score of 0). In half of the samples (28/52), T-cells (CD3) and B-cells (CD20) could be identified, suggesting diffuse infiltration and this was associated with intermediate VAS (9–70) and synovitis histological scores (0.5–5). In 10 cases formation of small lymphocyte aggregates could be observed (Fig. 1), VAS range 6–80, and synovitis scores 2.5–6. The presence of an ectopic germinal centre-like structure was only seen in one sample (VAS 72, synovitis score 8).

### Differences in immune cell composition between HC and OA

#### Lymphocyte lineages

**Fig. 2:**
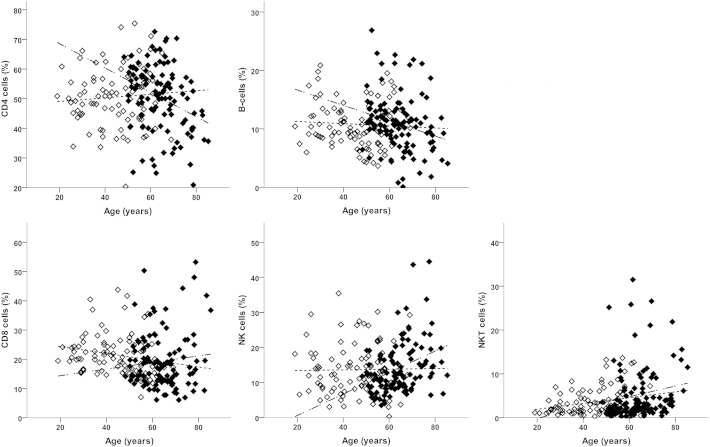
Lineage blood cell composition in HC and OA. Percentage of each cell subset lineage is represented as % total lymphocytes in HC (open symbols) and OA (black symbols). Lines depict significant age relationship as determined by regresion allowing age association to differ between HC and OA in individual subsets (see also Table II). The dotted line represents the relationship in HC only and dash-dot line in OA only.

When all immune cell lineages were considered simultaneously, there was an overall association with age (*P* = 0.008) but having adjusted for age there was no evidence of a difference between HC and OA (*F*_(4,195)_ = 1.14, *P* = 0.338). Assuming the association with age was the same in both groups, looking at the age-adjusted results for individual lineages (Table II), there were no differences in the levels of each lineage between HC and OA, consistent with the non-significant overall test. However, when an interaction term was added to the multivariate model, the association with age differed between OA and HC (*P* = 0.001; Fig. 2). This interaction remained statistically significant (*P* = 0.010) when the analysis was restricted to individuals aged 48 or above (the minimum age in the OA group).

Looking at individual lineages (Fig. 2), there was no association with age in HC for NK and NKT-cells, whilst their frequencies were higher in OA patients with increasing age. For CD4^+^ T-cells and B-cells there was no association in HC, but frequencies were lower in OA patients. For CD8^+^ T-cells the trends were in opposite directions, with older HCs having lower frequencies (negative trend) whilst older OA patients had higher frequencies (positive trend).

#### CD4^+^ T-cell phenotypes

Using the same strategy, proportions of naive, memory and IRC CD4^+^ T-cells considered simultaneously and having adjusted for age revealed no apparent difference between groups (*F*_(3,177)_ = 0.30, *P* = 0.824), and the association with age was significant when fixed in both groups (*F*_(3,177)_ = 7.56, *P* < 0.001).

Looking at each phenotype individually, consistent with the overall result and current knowledge^22^ frequencies did not differ between the HC and OA: naïve CD4^+^ T-cell frequencies reduced with age, memory CD4^+^ T-cell frequencies increased, whilst CD4^+^ IRC frequencies did not vary with age (Table II). There was a significant interaction with age in the multivariate analysis (*F*_(3,176)_ = 3.18, *P* = 0.026).

**Fig. 3:**
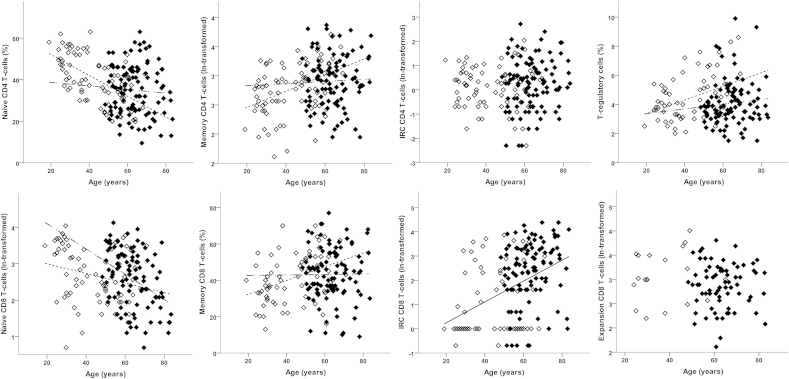
CD4+ and CD8+ T-cell subset phenotypes in HC and OA. Percentage of CD4+ T-cell (top) and CD8+ T-cell (bottom) subsets in HC (open symbols) and OA (black symbols). Lines depict significant age relationship with subset. The non-broken line represents the relationship when similar in HC and OA, dotted line for relationship in HC only and dash-dot line for OA only.

Looking at each CD4^+^ T-cell phenotype individually, known positive (memory) and negative (naïve) age associations were observed in HC (Table II and Fig. 3), but neither of these subsets was associated with age in OA patients.

With respect to CD4^+^ regulatory T-cells, having adjusted for age, the OA patients had comparatively fewer Treg than HC (Table II, Fig. 3). When the age effect was fixed in both groups, the frequency of Treg was higher in older individuals; however the association with age differed, being positive in HC but absent in OA patients (Fig. 3).

#### CD8^+^ T-cell phenotypes

Memory like CD8^+^ T-cells were not present in all individuals (*n* = 90 combing HC and OA) and were therefore analysed separately. These cells were more likely to be found in OA patients (71.7%; 71/99) than in HC (26.0%; 19/73) having adjusted for age [OR = 14.91 (5.40, 41.18); *P* < 0.001]. The odds of memory-like cells being present decreased with age [OR = 0.97 (0.94, 1.00); *P* = 0.037] but there was no evidence that this association differed between HC and OA (*P* = 0.233). In patients with memory-like cells present, the proportion of memory-like cells did not differ between HC and OA (Table II).

When frequencies of naive, memory and IRC CD8^+^ T-cells were assessed in simultaneous analysis, evidence of age-adjusted between-group differences (*F*_(3,167)_ = 8.30, *P* < 0.001) and an association with age (*F*_(3,167)_ = 10.02, *P* < 0.001) were observed.

Looking at individual phenotypes and having adjusted for age, OA patients tended to have more naïve and IRC CD8^+^ T-cells relative to HC (Table II). The proportion of memory CD8^+^ T-cells did not differ between groups. However, a significant interaction between age and group in the multivariate analysis (*F*_(3,166)_ = 3.67, *P* = 0.014) which persisted in patients aged 48 and over (*F*_(3,128)_ = 3.59, *P* = 0.016) suggested that the extent of between-group differences was not consistent at all ages.

Naive CD8^+^ T-cell frequencies reduced with age (Fig. 3) as expected in HC^22^ and in OA, but the negative trend with age was steeper in OA patients (Table II). In HC there was a positive association with age for memory CD8^+^ T-cell (as previously reported^22^), which was absent in OA patients (Fig. 3). Trends with age did not differ significantly for IRC or memory CD8^+^ T-cell.

It is worth noting that the proportion of CD8^+^ T-cell that were defined as IRC exceeded 1% in almost all of the OA patients (92.0%; 92/100) compared to only 38.7% (29/75) of the HCs. In the majority of HCs the proportion was at or around 1%, suggesting that this may be the ‘normal’ level in health. Using logistic regression to model the odds of IRC CD8^+^ T-cells being elevated above 1% in OA and controlling for age, odds were 14-fold higher (OR = 14.3; 95% CI 5.0 to 40.7; *P* < 0.001); there was no indication that the odds of elevated IRC were associated with age (*P* = 0.331) or that an age association differed by group (*P* = 0.848).

#### B-cell phenotypes

PPC were relatively rare and were investigated separately. The proportion of individuals with detectable PPC did not differ between HC (47%; 21/44) and OA (44%; 47/106) and after adjusting for age (OR = 0.95 (0.33, 2.71); *P* = 0.924). In people with PPC, there was a borderline-significant difference between the groups, with OA patients having relatively fewer PPC than HC (Table II). There was no evidence to suggest that PPC frequency was associated with age (*P* = 0.378) or that this (lack of) association differed between the groups (*P* = 0.990).

**Fig. 4:**
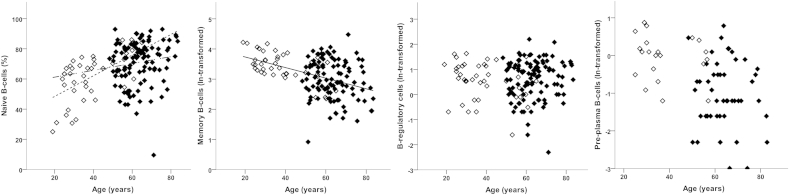
B-cell subset phenotypes in HC and OA. Percentage of each B-cell subsets in HC (open symbols) and OA (black symbols). Lines depict significant age relationship with subset. The non-broken line represents the relationship when similar in HC and OA, dotted line for relationship in HC only and dash-dot line for OA only.

Considering naive, memory and T2/Breg simultaneously, there was an association with age (*F*_(3,145)_ = 6.50, *P* < 0.001) but no evidence of differences between OA and HC (*F*_(3,145)_ = 0.55, *P* = 0.646). Looking at individual phenotypes, naive B-cell frequencies tended to be higher in older individuals (Fig. 4) whilst memory B-cell frequencies tended to be lower. T2/Breg frequencies did not vary with age (Table II, Fig. 4). There was evidence of an interaction between age and group in the multivariate analysis (*F*_(3,144)_ = 3.25, *P* = 0.024); however, only naïve B-cells showed a difference in the age association between the HC and OA. There was a relatively higher naïve-cell frequency with age (positive trend), but this association was weaker in OA to the extent that the confidence interval crossed 0.

### Clinical correlate to immune cell blood composition

In a subgroup of patients (with reported duration), longer OA duration was associated with the loss of CD4^+^ T-cells (*n* = 62, *rho* = −0.297, *P* = 0.025). In the 46 patients who reported a family history of OA, there was a more profound loss of total CD4^+^/CD8^+^ T-cells (*P* = 0.077). A trend between CD4^+^ IRC (but not CD8^+^) frequency and disease duration was observed (*n* = 62, *rho* = +0.248, *P* = 0.058).

For the majority of patients, we had data on the predominant joints diagnosed with OA (Table I). CD4^+^ IRC were more frequent in patients with hip (*P* = 0.002, median 1.3% (range 0.1–8.0) for no hip vs 2.9% (0.5–7.9) with hip) and foot (*P* = 0.023, median 1.1% (range 0.1–8.0) vs 2.3% (0.8–5.6) OA). Higher CD4^+^ IRC frequencies were also observed in patients with knee OA although with only 12 patients with no knee OA, significance was low (*P* = 0.300, median 0.8% (range 0.1–2.8) vs 2.0% (0.1–12.7). These data suggest that OA in large joints may be an important determinant of the presence of CD4^+^ IRC in the blood. We observed no further association between demographic and clinical parameters or anatomic OA localisation for the B-cell subsets/phenotypes.

## Discussion

Immunosenescence in the elderly encompasses many mechanisms resulting in compromised immune function and subsequent increased susceptibility to infection, cancer and autoimmune diseases. This novel, though preliminary, analysis of the immune cell composition of the blood of OA patients showed that several lineages and sub-phenotypes of T and B-cells were affected in OA beyond what appears directly related to ageing and may therefore reflect additional immune dysfunction related to the OA process itself. We proceeded to an age adjusted analysis that confirmed lack of age association in health for NK, CD4^+^ T-cells, B-cells and NKT cells but a trend for lower frequency for CD8^+^ T-cells with ageing. In OA, CD4^+^ T-cell and B-cell frequency were lower while CD8^+^ T-cell frequency was higher compared with HC. With respect to cell phenotyping, the main differences observed were associated with the CD8^+^ T-cells: memory-like cells were 15-fold more likely to be found in OA independent of age; reduction in naïve cell frequency with age was more accentuated in OA; while the relationship between age and memory CD8^+^ T-cells observed in health was lost in OA. The odds of increased IRC frequency were also much increased in OA compared to health. In CD4^+^ T-cells, the reduction in naïve and increase in memory and Treg subsets with age previously reported in HC^22^ were lost in OA. Finally B-cells showed evidence of a limited disturbance in the age relationship for naïve cells but no change for memory B-cells and T2/Breg, nor for the presence of PPC.

As a general comment, because the extent of the differences between groups could vary with age, interpreting the estimated between-group differences in cell frequency should be done with caution, as it is difficult to determine whether this is a true observation without longitudinal data, especially data including people who develop OA with time. Were these overall differences to be confirmed, this could be interpreted as a shift in overall blood composition in OA, but as the proportion of one subset/phenotype cannot change without affecting at least one other, it is difficult to identify which specific lineage/subset drives this difference between the groups. Despite these limitations, to our knowledge, these data are the first report that the immune cell composition of the blood of OA patients is affected beyond what would be expected during ageing.

In health, minor alterations in B lymphocytes were reported in ageing and the loss of cell-mediated responses was predominantly associated with alterations in the function of T-cells^24^. Accordingly, lineage representations were not strongly associated with age in health (with the exception of CD8^+^ T-cells), although clear changes were observed in particular T and B-cell sub-phenotypes. These data confirmed previous observations of thymic decline in generating naïve CD4^+^ T-cells and CD8^+^ T-cells with age as well as the development of memory subset reflecting T-cell homeostasis over a lifetime^22^. The increase in frequency of regulatory T-cells has been hypothesized^25^ but we only verified it by being able to recruit HC with a wide age range. B-cells continue to develop throughout life (releasing naïve cells into the blood stream) while memory cells developing into plasma cells tend to home to the bone marrow and therefore leave the blood. Accordingly our data showed increase and decrease of circulating naïve and memory B-cells respectively.

Previous data showing no differences for CD4^+^ or CD8^+^ T-cells frequencies were reported comparing OA with age-matched HC over 1 decade of age (mean age 70 range 65–75)^26^. Our data would probably have been consistent with these previous results if restricted to that decade of age. The relative changes of T-cells in OA (decline in CD4^+^ and absence of reduction of CD8^+^ T-cells) were only visible in our study because we included a wide range of age in HC. The decline in CD4^+^ T-cells may suggest greater loss in T-cell functionality in OA compare to health. How CD8^+^ homeostasis is controlled throughout ageing remains unclear. Clonal expansions of CD8^+^ T-cells related to Epstein Barr and/or CMV were shown to accumulate in ageing^27^. The presence of such expanded highly differentiated CD8^+^ T-cells was shown to be detrimental to immunity^28^, ^29^, ^30^ resulting in the increased morbidity and mortality of the elderly population. The presence in almost all OA patients of greatly expanded CD8^+^ T-cells with an IRC or the memory-like phenotype may reflect an even greater disturbance of CD8^+^ homeostasis in OA.

The relationship between NKT and age in HC was maintained in OA. In a few OA patients, however, very high frequencies were observed (Fig. 2). The role of the NKT population of cells expressing both T-cell and NK-cell markers is not well understood, but they functionally work both like NK and T-cells, notably by immediately producing cytokines when activated (interferon-gamma, tumour necrosis factor-alpha and IL-2)^31^, ^32^. NKT-cells are therefore part of the first-line defense mechanism against pathogens including viruses. One of their functions associated with ageing is to compensate for the deterioration of T-cells and although NKT production of IFN-gamma also decreased with ageing^33^ it is better maintained (notably in centenarian) and is thought to be essential for healthy ageing. Inflammation in metabolic tissues has emerged as a universal feature of obesity and its co-morbidities, including OA. A link between diet and NKT frequencies has also been established^34^ and mice lacking NKT were more prone to obesity and inflammation^35^. In OA, patients with high NKT-cell frequencies may therefore suggest different capacities for immediate response to viral infection.

Phenotyping CD4^+^ T-cells revealed further differences. We observed a lower frequency of regulatory T-cells, and higher than expected naïve CD4^+^ T-cells. This last observation was intriguing as it is quite similar to what is observed in RA where such maintenance of high naïve T-cells is predictive of good outcome^36^. IRC have been shown to develop as a result of pro-inflammatory cytokine stimulation^22^. In our study, IRC were more frequent in patients with large joint OA (hip, knee, foot). It could therefore be speculated that their presence reflects a greater volume of synovitis. Unfortunately we did not have whole body radiographs in order to fully characterise both the total-body burden and the joint-level severity of OA in individuals. The reduction of regulatory T-cells in the blood of OA is also intriguing and this has never been reported to our knowledge. Such loss of Treg (notably as Treg were shown to accumulate with ageing) may have important consequences. Autoantibodies are both a feature of ageing and OA and, although a direct role for B-cell auto-reactivity remains a matter of debate in OA, there is much evidence of accumulated auto-reactivity, notably towards cartilage epitopes (with autoantibody to collagens, proteoglycans)^37^. Loss of regulatory mechanisms may be contributing to the loss of tolerance resulting in such autoantibodies being generated^38^. The loss of relationship between total and naïve B-cell subset and age in OA is not sufficient to suggest causality although it may suggest that additional disease effect may be mediated by B-cells, especially considering the highly differentiated state of synovial B-cells^39^.

The histology and immunohistochemistry analysis of OA synovial biopsies was primarily used to confirm previously reported data related to the OA synovial membrane cellular composition. T and B-cell infiltration was observed and even if aggregates were not as frequently observed as in RA, their presence in the synovial tissue suggests immune-related events in OA. Synovitis score were above normal in all but 13 patients and reached quite high levels. Taken together with the changes seen in blood cell composition, our data suggest that immune dysregulation may contribute to OA in a way that remains to be fully elucidated.

In conclusion, our data suggest that alterations in the blood immune cell composition can be observed in OA, and such changes appear different to those associated with ageing alone. Some of the changes may reflect inflammation and autoreactivity. The loss of regulatory mechanisms in OA should therefore be further investigated in relation to auto-antibodies and auto-reactivity development. Future research into immune-modulation for the treatment of OA may be of benefit.

## Authors contribution

FP Conception and design, analysis and interpretation of the data; FP, AB Collection and assembly of data; EH statistical analysis, RR, MC Provision of study materials or patients; FP, AB, EH, PC Drafting of the article; FP, PE, PC Critical revision of the article for important intellectual content; AB Administrative, technical, or logistic support. All authors approved final version of this manuscript.

## Funding source

This work was supported by the IMI-funded project BeTheCure (No 115142-2), grant and Arthritis Research UK grants: 19545 (HERO) and 20083 (Experimental Osteoarthritis Treatment Centre). RR is funded by the Rose Hellaby Scholarship (Guardian Trust) New Zealand, The Royal Australasian College of Physicians (RACP)/Australian Rheumatology Association & Starr Fellowship (Australia), New Zealand Rheumatology Association and Health Workforce New Zealand (contract number 242815/347837/00). MC was holder of University of Ottawa International Traveling Fellowship (#31300). FP, PE and PC are part-funded by the National Institute for Health Research (NIHR) through the Leeds Musculoskeletal Biomedical Research Unit.

## Conflict of interest

Authors declare that there is no conflict of interest associated with this publication.

This article presents independent research funded by the NIHR. The views expressed are those of the authors and not necessarily those of the NHS, the NIHR or the Department of Health.

## Figures and Tables

**Table I:** Descriptive characteristics of the studied groups

**HC (n** = **121)**	
Mean age in years(SD), range	44.0 (12.7), 19-71
Sex M/F	37/85
**OA biopsies (n** = **52)**	
Mean age in years(SD), range	57 (13.5), 30-89
Sex M/F	32/82
**OA (n** = **114)**	
Mean age in years(SD), range	64 (10.9), 48-85
Sex M/F	32/82
Mean BMI, (range)∗	30 (21–50)
Mean symptom duration in years, (range)∗	10 (1–35)
Family history (n)∗	46
Anatomical localisation (n)†	
knee	76
hip	30
hand	32
foot	13
ankle	2

∗ BMI was available for 96 OA patients, while symptom duration and family history were recorded for 62 patients.

† Some patients had OA in more than one joint area.

**Table II:** Differences in immune cell relative frequencies between HC and OA patients obtained from multivariate analyses

	Age-adjusted results assuming association with age is the same in HC and OA						Allowing age association to differ between HC and OA		
HC	OA	Difference between groups		Association with age		HC	OA	Interac-tion
Mean (95% CI)	*P* value	Change per year (95% CI)	*P* value	Change per year (95% CI)	Change per year (95% CI)	*P* value
**Lymphocyte lineage** Age mean, SD (range)	**n** = **93** 44.8, 12.1 (19–69)	**n** = **108** 64.2,8.9 (48–85)							
NK, mean %	14.92	12.97	−1.95 (−4.70, 0.79)	0.162	0.12 (0.03, 0.22)	0.011	0.01 (−0.11, 0.13)	0.31 (0.16, 0.46)	0.003
NKT, mean %	4.47	4.49	0.02 (−1.88, 1.91)	0.987	0.09 (0.02, 0.15)	0.010	0.07 (−0.02, 0.15)	0.12 (0.02, 0.23)	0.025
CD8, mean %	21.16	19.62	−1.54 (−4.78, 1.69)	0.348	−0.03 (−0.14, 0.09)	0.632	−0.12 (−0.26, 0.03)	0.11 (−0.07, 0.29)	0.051
CD4, mean %	49.29	51.53	2.24 (−1.76, 6.24)	0.271	−0.12 (−0.26, 0.02)	0.087	0.06 (−0.12, 0.23)	−0.41 (−0.63, −0.19)	0.001
B-cells, mean %	10.16	11.40	1.23 (−0.45, 2.92)	0.149	−0.06 (−0.12, 0.00)	0.038	−0.02 (−0.09, 0.06)	−0.13 (−0.23, −0.04)	0.066
**CD4 T-cells** Age mean, SD (range)	**n** = **81** 43.1, 13.4 (19–69)	**n** = **101** 63.8, 8.9 (48–83)							
Naïve, mean %	36.44	38.19	1.75 (−2.74, 6.25)	0.443	−0.35 (−0.50, −0.21)	<0.001	−0.50 (−0.68, −0.32)	−0.09 (−0.33, 0.16)	0.008
Memory, mean %	17.98∗	16.79∗	0.93 (0.78, 1.11)†	0.444	0.97 (0.39, 1.55)‡	0.001	1.40 (0.68, 2.12)‡	−0.24 (−1.80, 1.34)‡	0.048
IRC, mean %	1.19∗	1.27∗	1.07 (0.73, 1.57)†	0.723	0.41 (−0.85, 1.70)‡	0.520	N/A	N/A	0.167
**Regulatory T-cells** Age mean, SD (range)	**n** = **104** 41.8, 12.7 (19–69)	**n** = **114** 63.9, 9.0 (48–83)							
Treg, mean %	4.84	3.37	−1.17 (−1.72, −0.63)	<0.001	0.04 (0.02, 0.06)	<0.001	0.05 (0.03, 0.08)	0.02 (−0.01, 0.05)	0.073
**CD8 T-cells** Age mean, SD (range)	**n** = **73** 43.9, 13.8 (19–71)	**n** = **99** 63.87 8.9 (48–83)							
Naïve, mean %	11.52∗	15.81∗	1.37 (1.01, 1.86)†	0.041	−2.08 (−3.06, −1.09)‡	<0.001	−1.31 (−2.54, −0.07)‡	−3.41 (−5.00, −1.80)	0.042
Memory, mean %	44.13	41.16	−2.97 (−8.59, 2.67)	0.300	0.25 (0.06, 0.43)	0.010	0.38 (0.15, 0.61)	0.01 (−0.30, 0.32)	0.064
IRC, mean %	3.19∗	9.10∗	2.85 (1.68, 4.85)†	<0.001	2.03 (0.25, 3.84)‡	0.025	N/A	N/A	0.518
	**n** = **19**	**n** = **71**							
Expansion if present, mean %	22.03∗	17.55∗	0.80 (0.55, 1.16)†	0.228	−0.09 (−0.97, 1.17)‡	0.862	N/A	N/A	0.869
**B-cells** Age mean, SD (range)	**n** = **44** 40.4, 12.9 (31–65)	**n** = **106** 62.1, 8.8 (48–83)							
Naïve, mean %	69.36	67.87	−1.49 (−8.89, 5.91)	0.691	0.45 (0.23, 0.68)	<0.001	0.69 (0.37, 1.00)	0.23 (−0.08, 0.54)	0.044
Memory, mean %	22.22∗	21.99∗	0.99 (0.73, 1.34)†	0.946	−1.72 (−2.62, −0.81)‡	<0.001	N/A	N/A	0.260
Breg, mean %	1.63∗	1.93∗	1.18 (0.79, 1.77)†	0.412	−0.38 (−1.59, 0.84)‡	0.535	N/A	N/A	0.179
	**n** = **21**	**n** = **47**							
Pre-plasma if present, mean %	0.80∗	0.40∗	0.50 (0.25, 1.00)†	0.050	−0.94 (−3.01, 1.18)‡	0.378	N/A	N/A	0.990

∗ Geometric mean.

† Ratio OA: HC.

‡ Geometric mean percentage change per year.
