# Supplementary material for: Changes in peripheral blood immune cell composition in osteoarthritis
Source: Osteoarthritis Cartilage. 2015 Nov;23(11):1870–8. doi: 10.1016/j.joca.2015.06.018 (PMC4638189; doi:10.1016/j.joca.2015.06.018)
Supplement: Supplementary file 1 [file mmc1.docx]

**Supplementary Material**

**Changes in peripheral blood immune cell composition in osteoarthritis.**

Frederique Ponchel^1^, Agata Burska^1^, Elizabeth MA Hensor^1^, Rafi Raja^1&^, Mark Campbell^1#^, Paul Emery^1^, Philip G Conaghan^1^

^1^ Leeds Institute of Rheumatic and Musculoskeletal Medicine, University of Leeds & NIHR-Leeds Musculoskeletal Biomedical Research Unit, Leeds, UK

Table S1: Flow cytometry clones descriptive.

| marker | clone | fluorochrome | company |
| --- | --- | --- | --- |
| **Lineage panel**  CD3  CD4  CD8  CD56  CD19  CD16  CD14 | UCHT1  RPA-T4  RPA-T8  B159  HIB19  3G8  M5E2 | FITC  V500  PERCP5  PE  BV421  PE-CY7  APC | BD  BD  BD  BD  Biolegend  BD  BD |
| **T-cell panel**  CD3  CD4  CD45RB  CD45RA  CD45RO  CD62L | UCHT1  RPA-T4  MEM-55  F8-11-13  UCHL1  145/15 | V500  BV421  FITC  PE  Alexa700  APC | BD  BD  SEROTEC  SEROTEC  BD  MILTENYI |
| **Treg panel**  CD3  CD4  CD25  FOXp3  CD127 | UCHT1  RPA-T4  2A3  236A/E7  HIL-7R-M21 | V500  BV421  PE CY7  ALEXA488  PERCP-CY5.5C | BD  BD  BD  BD  BD |
| **B-cell panel**  CD19  CD38  CD27  CD24 | HIB19  HB7  M-T271  ML5 | BV421  PE-Cy7  PE  FITC | Biolegend  BD  BD  BD |


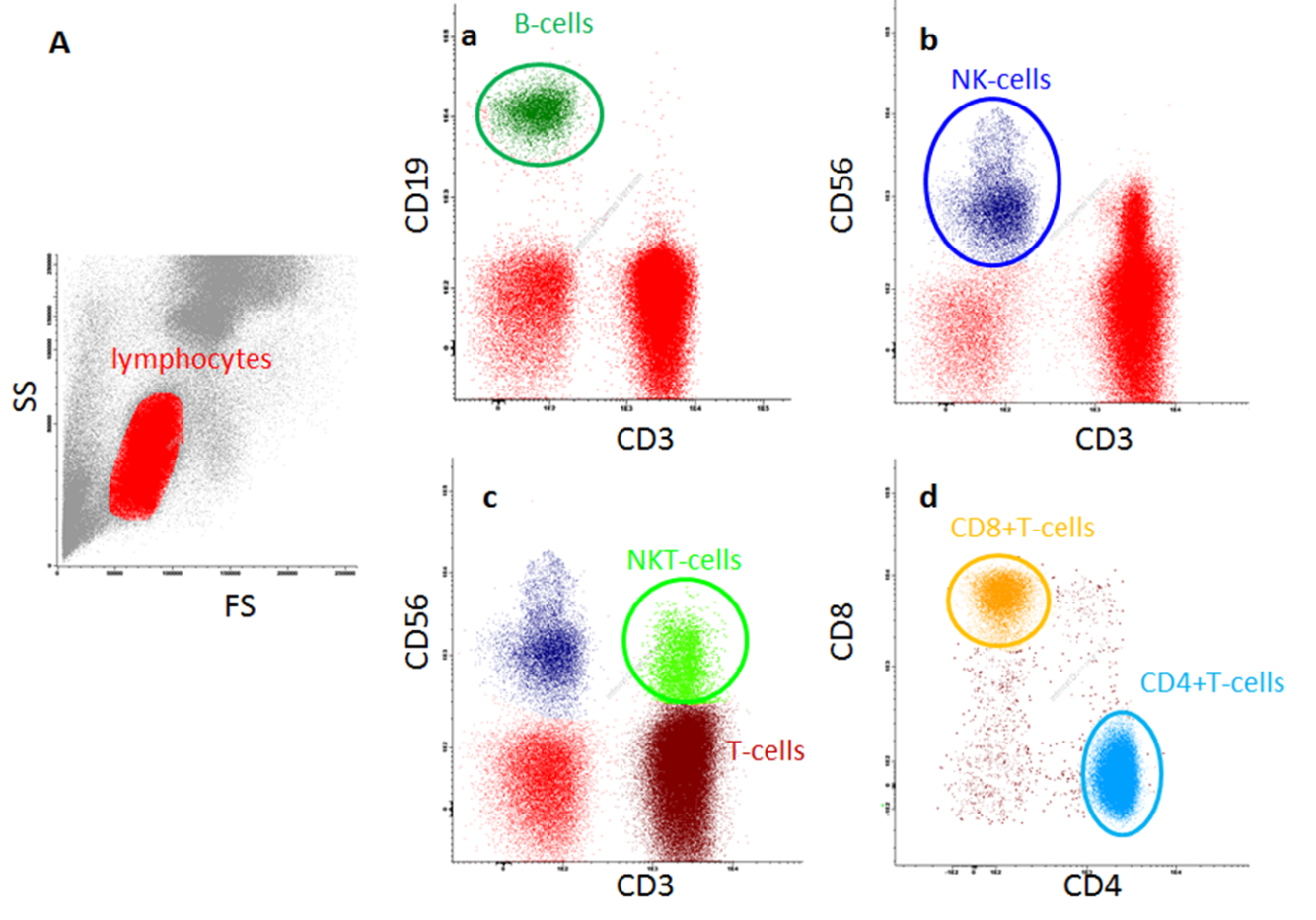
Figure S1. Gating strategies for lineage and subset phenotype.

A)

B)


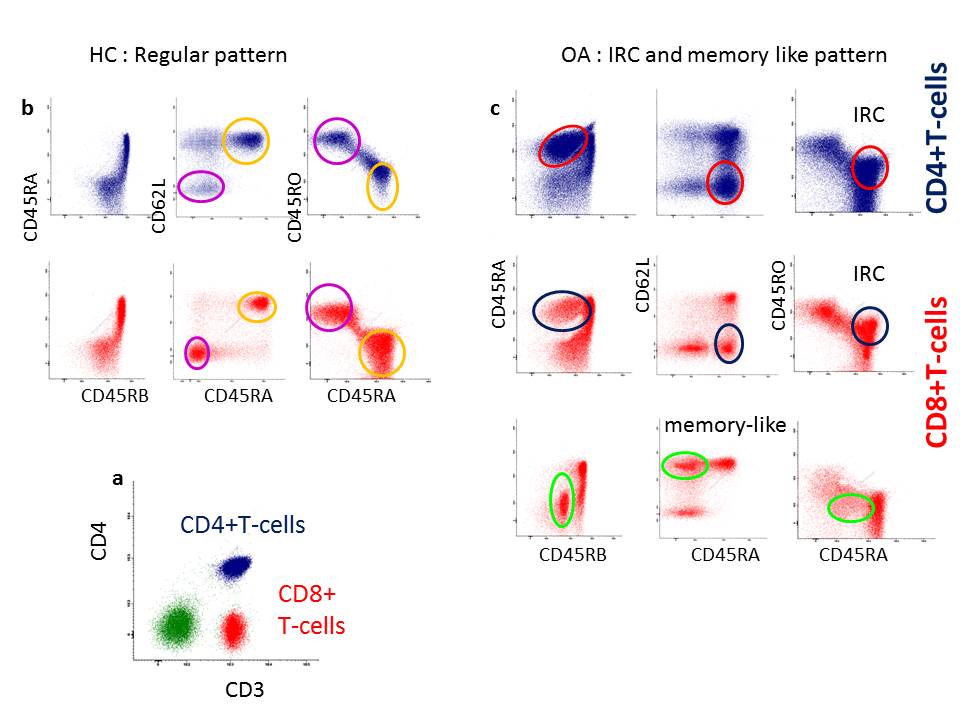


C)


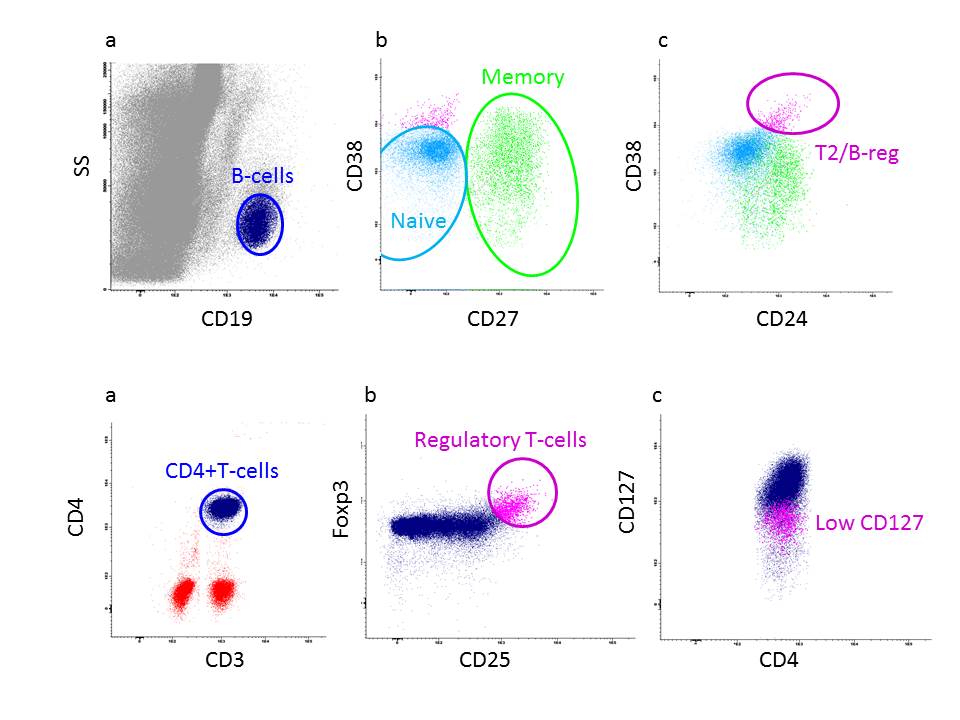


D)


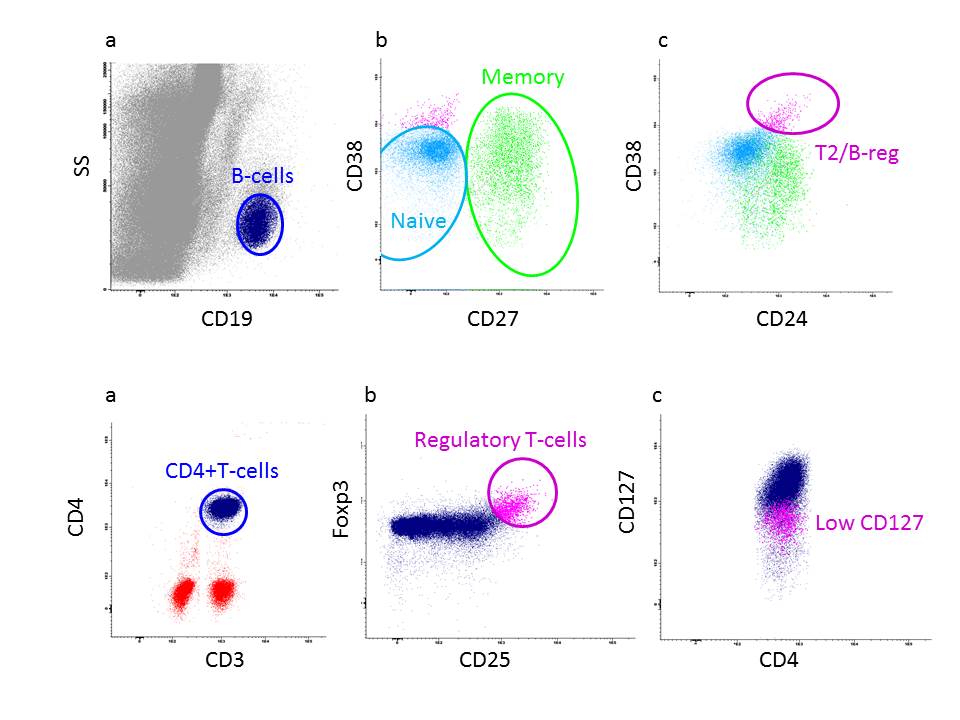


1. Lymphocytes were gated on forward and scatter parameters. B-cells were first detected using CD19 expression (panel a, dark green, 11.5%). NK-cells were then gated on a dual plot for CD3 and CD56 (panel b, dark blue, 12.7%). NKT-cells were then identified as double CD3^+^/CD56^+^ (panel c, light green, 5.1%). T-cells (identified as CD3+CD56-, brown) were further separated as CD4 (panel light blue, 58.9%) and CD8 T-cells (orange, 11.8%).

B) CD4 and CD8 T-cells were first gated (panel a).Representative flow cytometry dual plot showing phenotypes in CD4 (blue plots) and CD8 (red plots) T-cells using the expression of the CD45-RB-RA and RO with CD62L markers. A typical panel is presented for a HC (panel b) and abnomal phenotypes observed in 2 OA patients (panel c). In both CD4 and CD8 T-cell naïve cells (orange circle, 55% and 49% respectively) and memory cells (pink circles, 28% and 39% respectively) are presented in panel b. The IRC in CD4^+^ (red circles, 28% in the OA patient) and CD8^+^ IRC (blue circles, 31% in the OA patient) as well as the memory like expansion in CD8^+^ T-cells (green circles, 23% in the OA patient) are examplified in panel c, in two different OA patients as both are rarely observed simultaneously.

C) B-cells were gaited on expression of CD19 (panel a). Naïve (71%) and memory B-cells (23%) were identified based on the expression of CD27 and CD 38 (panel b). Putative T2/Breg (3.2%) were gated using high expression of CD38 and CD24 (panel c).

D) CD4^+^ T-cells were gated (panel a). The high expression of CD25 and of FoxP3 was used to identify Treg population (panel b, 6.7%). The gate was refined to ensure no Treg cells were expressing high levels of CD127 (panel c).
